# Environmental and Climatic Drivers of Phytoplankton Communities in Central Asia

**DOI:** 10.3390/biology13090717

**Published:** 2024-09-12

**Authors:** Fangze Zi, Tianjian Song, Jiaxuan Liu, Huanhuan Wang, Gulden Serekbol, Liting Yang, Linghui Hu, Qiang Huo, Yong Song, Bin Huo, Baoqiang Wang, Shengao Chen

**Affiliations:** 1 College of Life Sciences and Technology, Tarim Research Center of Rare Fishes, Tarim University, Alar 843300, China; 10757213076@stumail.taru.edu.cn (F.Z.); ljx18054379101@126.com (J.L.); whh18699866806@126.com (H.W.); 10757231098@stumail.taru.edu.cn (G.S.); 10757231127@stumail.taru.edu.cn (L.Y.); 10757222068@stumail.taru.edu.cn (L.H.); 10757223082@stumail.taru.edu.cn (Q.H.); 120050013@stumail.taru.edu.cn (Y.S.); 2 College of Water Sciences, Beijing Normal University, Beijing 100875, China; 202131470020@mail.bnu.edu.cn; 3 State Key Laboratory of Environmental Criteria and Risk Assessment, Chinese Research Academy of Environmental Sciences, Beijing 100012, China; 4 College of Fisheries, Huazhong Agricultural University, Wuhan 430070, China; huobin@mail.hzau.edu.cn; 5 Institute of Hydrobiology, Chinese Academy of Sciences, Wuhan 430072, China; wangbq@ihb.ac.cn

**Keywords:** phytoplankton diversity, artificial water bodies, topographic barriers, Central Asia

## Abstract

**Simple Summary:**

This study examines the influence of topographic barriers on phytoplankton diversity in artificial water bodies in Central Asia. By analyzing water samples from 14 locations across the Altai and Tianshan mountains, we discovered that topographic features significantly impact environmental conditions such as water temperature and nutrient levels. These ecological differences lead to variations in the phytoplankton community structure, with areas of more complex topography supporting higher diversity (valley forests, wetlands, deserts, etc.). Our findings highlight the importance of considering topographic factors in managing and conserving water resources in the region.

**Abstract:**

Artificial water bodies in Central Asia offer unique environments in which to study plankton diversity influenced by topographic barriers. However, the complexity of these ecosystems and limited comprehensive studies in the region challenge our understanding. In this study, we systematically investigated the water environment parameters and phytoplankton community structure by surveying 14 artificial waters on the southern side of the Altai Mountains and the northern and southern sides of the Tianshan Mountains in the Xinjiang region. The survey covered physical and nutrient indicators, and the results showed noticeable spatial differences between waters in different regions. The temperature, dissolved oxygen, total nitrogen, and total phosphorus of artificial water in the southern Altai Mountains vary greatly. In contrast, the waters in the northern Tianshan Mountains have more consistent physical indicators. The results of phytoplankton identification showed that the phytoplankton communities in different regions are somewhat different, with diatom species being the dominant taxon. The cluster analysis and the non-metric multidimensional scaling (NMDS) results also confirmed the variability of the phytoplankton communities in the areas. The variance partitioning analysis (VPA) results showed that climatic and environmental factors can explain some of the variability of the observed data. Nevertheless, the residual values indicated the presence of other unmeasured factors or the influence of stochasticity. This study provides a scientific basis for regional water resource management and environmental protection.

## 1. Introduction

In ecology, topographic barriers usually refer to natural features in the geographic environment, such as mountains, rivers, lakes, and other geomorphological features [1,2]. They may impact the distribution, migration, and population dynamics of organisms [3]. Topographic barriers can separate ecosystems in different areas, causing groups of organisms to evolve into different subspecies or species on either side of the topographic barrier [4]. Such isolation can act as a barrier to migratory animals’ migration routes, leading to the evolutionary independence of populations within each silo, which increases localized species diversity and affects the population structure and distribution of species [5]. Furthermore, topographic barriers may impose different natural selection pressures on groups of organisms as they adapt to environmental conditions, positively or negatively affecting biodiversity in other contexts, promoting adaptive evolution at the species level [6,7].

Phytoplankton, which are mainly tiny plants suspended in water bodies, are ecologically crucial in ecosystems [8,9]. Phytoplankton are essential food sources for many aquatic organisms, including zooplankton, copepods, and small fish [10]. They form the base of the marine food chain and support higher-level biomes. While phytoplankton can contribute to nutrient cycling and help maintain ecosystem balance, excessive growth of certain species can lead to harmful algal blooms, which can degrade water quality [11,12]. Phytoplankton are indispensable in the global carbon cycle, as they absorb carbon dioxide, help mitigate the effects of greenhouse gases, and impact climate regulation [13,14]. Overall, phytoplankton are crucial in aquatic ecosystems and are ecologically important for biodiversity, climate regulation, and water quality maintenance in water bodies.

Topographic barriers can impact phytoplankton distribution because they may affect environmental factors such as water movement and light [15,16]. Topographic barriers such as mountains or islands may impede the flow of the water column, resulting in different flow patterns on either side of the barrier [17]. This may affect the distribution of phytoplankton, resulting in different hydrodynamic conditions on either side of the water [18]. Mountain ranges may block sunlight at certain times, causing the back side of the range to be relatively shaded. This difference in light may affect the photosynthetic activity and distribution of phytoplankton [19]. Rivers passing through mountain ranges may experience temperature gradients at different heights [20]. The growth and distribution of phytoplankton are usually closely related to the temperature of the water column, so topographic barriers may indirectly affect the distribution of phytoplankton [21]. Mountain ranges may affect the movement of dissolved and suspended substances in rivers, influencing phytoplankton growth conditions [22,23]. Moreover, steep changes in water depth may occur in water bodies near mountain ranges, which may impact phytoplankton distribution and adaptation [24].

Artificial water bodies are artificial bodies constructed by humans on natural rivers or streams through engineering means such as dams or barrages [25]. They are usually used for water storage, water flow regulation, water supply, power generation, flood control, and ecological protection [26]. The construction of artificial lakes and reservoirs often requires full consideration of factors such as topography, water resources, and the environment and can impact the surrounding ecosystem and socioeconomy [27]. Phytoplankton communities in artificial water bodies are closely related to topographic barriers [28]. Topographic barriers such as mountains and hills create environmental separation in water bodies, altering flow dynamics and creating diverse habitats [29]. Such topographic obstacles lead to the formation of circulation patterns in the water body, which affect nutrient transport and distribution and, consequently, phytoplankton growth and distribution [30]. Topographic barriers promote the diversity and abundance of phytoplankton communities due to the different environmental conditions of water bodies in various regions [31]. Therefore, when studying and managing artificial lakes/reservoirs, it is essential to consider the impact of topographic barriers on watershed ecosystems to maintain the ecological balance of water bodies.

Research on phytoplankton communities in reservoirs in China has been gaining attention in recent years. Vinçon-Leite et al. developed a model to forecast cyanobacteria blooms in the Yuqiao Reservoir [32]. Yang et al. found that mixing regime, water temperature, and light availability were the key factors determining phytoplankton dynamics in an estuary reservoir in the Yangtze River [33]. Peng et al. investigated the viral infection characteristics of phytoplankton during cyanobacterial bloom succession in Xiangxi Bay [34]. Wang et al. identified odorants and algae species contributing to fishy odor occurrence in a reservoir in North China [35]. These studies collectively contribute to our understanding of phytoplankton in reservoirs in China, addressing various aspects such as distribution, succession, nutrient limitation, and management strategies. In this study, we found significant differences in phytoplankton diversity in the studied water bodies through community analysis and correlation analysis, with higher phytoplankton diversity in areas with more complex topographic features, which favored nutrient mixing and light penetration. These results suggest that it is important to consider topographic factors when understanding the distribution and ecological functions of phytoplankton in different waters.

## 2. Materials and Methods

### 2.1. Study Area

Xinjiang is the largest administrative region in Central Asia within the People’s Republic of China. Its complex geography encompasses vast deserts, grasslands, mountains, and lakes. The Altai Mountains and the Tianshan Mountains are two critical geographical features of Xinjiang. The Altai Mountains, located in northern Xinjiang, border Mongolia and Russia and are part of Central Asia. The Altai Mountains consist of transverse ridges that span China, Mongolia, and Russia. Its peaks, valleys, and lakes form a unique topography. The climate and hydrology of the Altai Mountains greatly impact northern Xinjiang, creating distinct climatic and ecosystem patterns on either side of the range. Located in central Xinjiang, the Tianshan Mountains are one of the world’s longest mountain ranges, running east–west and dividing the region into northern and southern parts. Their peaks and valleys create a diverse topography in the Xinjiang region.

**Figure 1:**
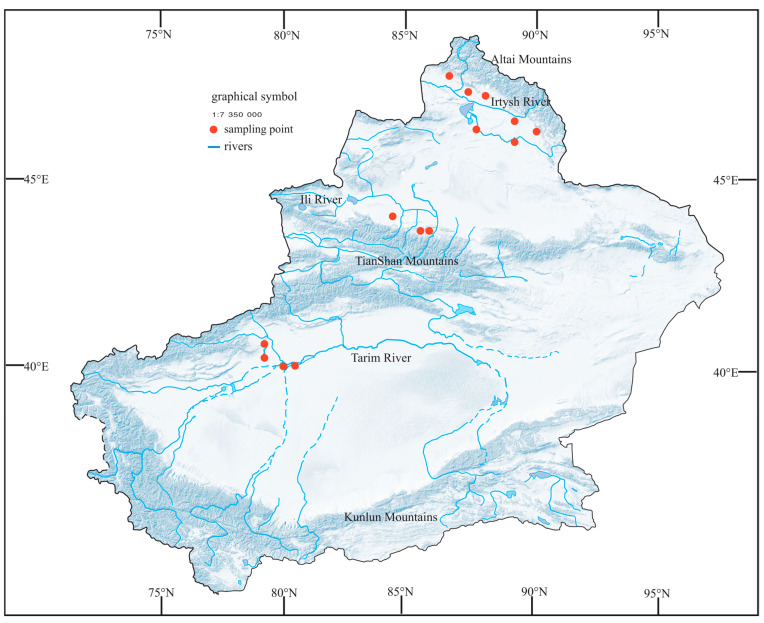
Map of the study site showing the location of the sampling sites.

Considering the exceptional geographic environment of the Xinjiang region, we selected 14 representative artificial water bodies for phytoplankton collection from May to July, 2021–2023, on the southern side of the Altai Mountains and the northern and southern sides of the Tianshan Mountains (Figure 1). Two sets of parallel samples were collected in the study area.

### 2.2. Sample and Environmental Data Collection

We collected 2 L of water samples filtered through a 25# plankton net (net length: 50 cm; net ring inner diameter: 20 cm; net coat: aperture 0.064 mm; PTN-S200 Purity, Beijing China) for collection, fixed in the field with 1% Ruggier’s solution (Solarbio, Beijing, China), brought back to the laboratory, and left for 36 h, then concentrated by siphoning to 50 mL for quantitative analysis [36]. According to the *Analytical Methods for Water and Wastewater Monitoring* (4th edition) [37], a plexiglass water collector was used to collect 1 L of water sample for subsequent testing. In addition, water samples were collected for the determination of total phosphorus (TP, mg/L) by ammonium molybdate spectrophotometry and total nitrogen (TN, mg/L) by alkaline potassium persulfate digestion UV spectrophotometry [37]. We took 0.1 mL in the laboratory and placed it in a phytoplankton counting frame for species identification and cell counting under a microscope (Olympus CX21) at 400×. Phytoplankton were identified in *Freshwater Algae: Identification, Enumeration and Use as Bioindicators* [38].

Water temperature (WT), pH, dissolved oxygen (DO), total dissolved solids (TDS), conductivity (C), salinity (SAL), and oxidation–reduction potential (ORP) were measured in situ using a Multi-Parameter Water Quality Sonde (YSI 556MPS). GPS data were used to determine each station’s coordinates (latitude and longitude); then, 19 layers of variables scaled to 30 s of meteorological data were downloaded from Wordclim (http://wordclim.org, accessed on 25 July 2024). Finally, meteorological factors were extracted in ArcGIS based on the latitude and longitude of each station. One meteorological factor could be extracted for each layer. The codes and specific descriptions of the 19 extracted meteorological variables are shown in the Supplementary Information.

### 2.3. Data Analysis

A Venn diagram was generated to analyze the differences in the abundances of shared phytoplankton between samples; differences between sample groups were determined using the non-metric multidimensional scaling (NMDS) method. Nonparametric similarity tests between groups of high-latitude data were performed using analysis of similarities (ANOSIM). A distance measure (or similarity) between objects was calculated from the variables, a relationship ranking was calculated, and a permutation test was performed on the rankings to determine whether the between-object group differences were much different from the within-group differences.

Phytoplankton diversity was analyzed using the Shannon–Wiener diversity index, Simpson’s diversity index, and Pielou uniformity index, which were calculated as follows:

Shannon–Wiener index H′ = –∑(N_i_/N)log2(N_i_/N)

$$\text{Simpson’s}\;\mathrm{diversity}\;\mathrm{index}\;D=1-\sum_{Ni}^{S}P_{i}^{2}$$

Pielou index J = H′/log2S

where N_i_ is the number of individuals of species i, N is the total number of individuals of all species, and S is the number of species.

Variation partitioning, or variance decomposition, is used to explain a situation in which two or more groups of explanatory variables belonging to different categories jointly explain a set of response variables and can effectively quantify the variance explained by two or more groups of variables individually and jointly.

ANOVA was performed using SPSS 25, NMDS was plotted using PRIMER 5.0 software, ANOSIM was statistically and graphically performed using the R package vegan, Venn diagrams were plotted using the R package given, and image manipulation was performed using Adobe Illustrator CC 2019. The other analyses were statistically and analytically performed using Excel 2010. To satisfy the assumption of multivariate normality and avoid extreme data effects, the data were log_(x+1)_-transformed before statistical analysis.

## 3. Results

### 3.1. Distribution Characteristics and Correlation of Water Environment Factors

**Figure 2:**
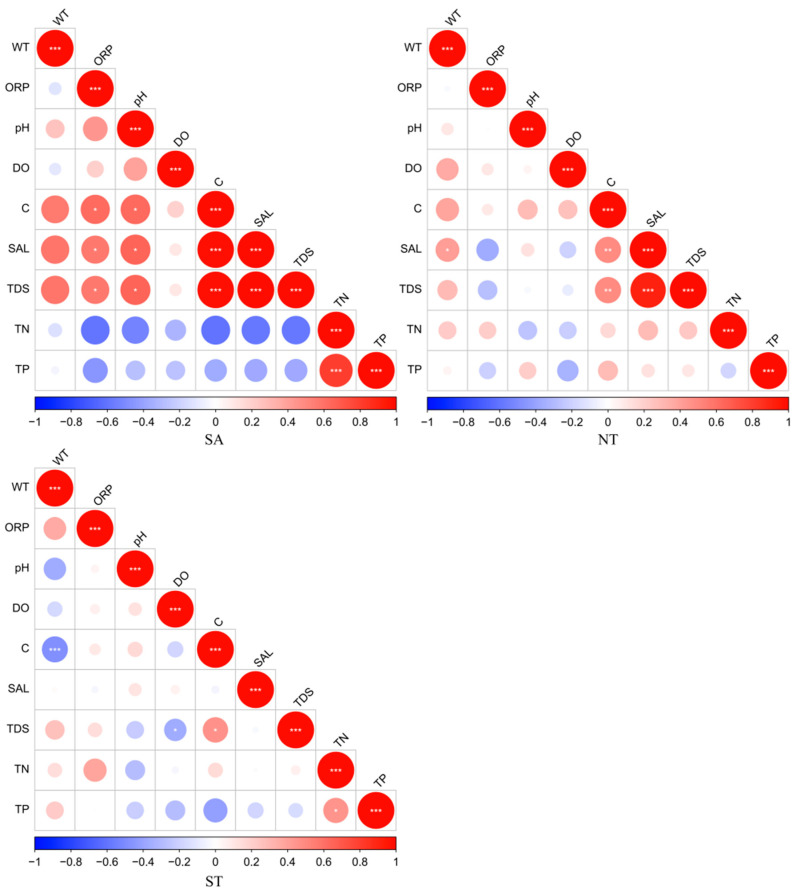
Correlation analysis of water environment parameters, red indicates a positive correlation and blue indicates a negative correlation, * Benjamini-Hochberg adjusted 0.01 ≤ *p* < 0.05; ** Benjamini-Hochberg adjusted 0.001 ≤ *p* < 0.01; *** Benjamini-Hochberg adjusted *p* < 0.001.

The results of this study, which pertain to the artificial water environment in Central Asia, showed that water environment indicators, such as physical (WT, ORP, pH, DO, C, SAL, TDS) and nutrient indicators (TN, TP), exhibited spatial variations to some extent. In the southern Altai Mountains, the physical indicators showed high correlations for C with SAL and TDS, and the nutrient indicators showed a high correlation for TN and TP in the northern Tianshan Mountains. In the southern Tianshan Mountains, C and WT were highly significantly related to the physical indicators, and TN and TP were significantly correlated with the nutrient indicators (Figure 2).

From the climatic data (http://wordclim.org, accessed on 25 July 2024), it could be seen that environmental factors such as the mean annual temperature, temperature of the hottest and coldest months, annual precipitation, and seasonal variations significantly affected phytoplankton growth and reproduction. The lowest yearly mean temperature of 4.08 °C was recorded in the southern Altai Mountains (later abbreviated SA). A mean annual temperature of 11.34 °C was recorded in the southern Tianshan Mountains (later abbreviated ST), and that in the northern Tianshan Mountains (later abbreviated NT) was 8.50 °C, which was relatively warm and more favorable for phytoplankton growth. The maximum temperature in the hottest month was 33.13 °C in the ST, 32.16 °C in the NT, and 28.98 °C in the SA. The minimum temperatures of the coldest month were −22.46 °C in the SA, −19.41 °C in the NT, and −14.93 °C in the ST. The SA had the highest annual precipitation with 175.15 mm, while the NT and the ST had annual precipitations of 144.25 mm and 51.75 mm, respectively. Precipitation in the wettest month was 27.31 mm in the SA and 20.75 mm in the NT, reaching 11.50 mm in ST. The seasonal variation in precipitation was greatest in the ST site at 75.01, whereas the NT (45.38) and SA (38.08) sites experienced relatively stable precipitation. The precipitation in the wettest season was 63.15 mm in the SA and 59.85 mm in the NT, reaching 29.50 mm in the ST. However, the precipitation in the driest season was 21.85 mm in the SA, 16.60 mm in the NT, and only 2.25 mm in the ST (Table 1).

### 3.2. Differences in Phytoplankton Community Structure across Geographic Regions

**Figure 3:**
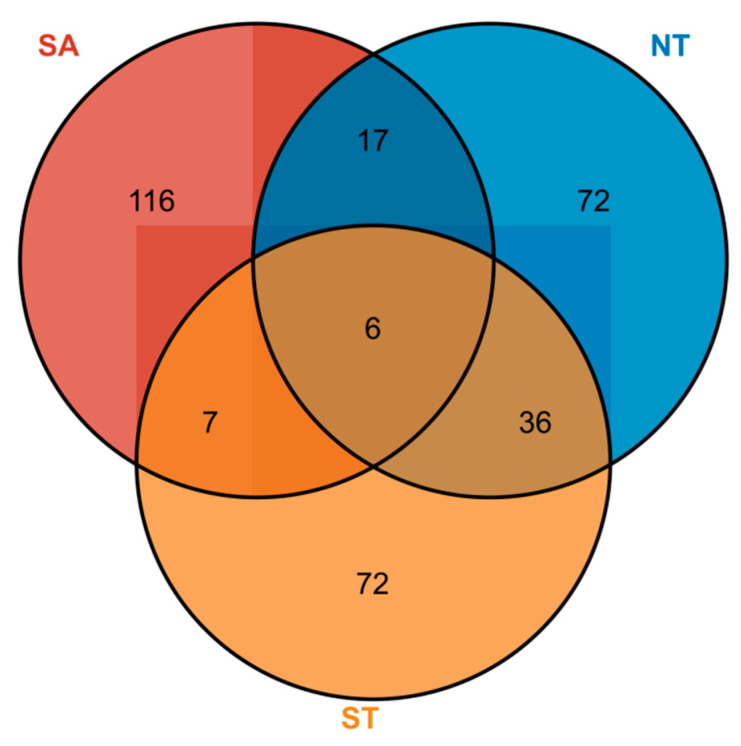
Phytoplankton species distribution in different geographical settings, Numbers in circles represent the number of phytoplankton species, SA for the southern Altai Mountains, ST for the south of Tianshan Mountains, and NT for the northern Tianshan Mountains..

A total of eight phytoplankton families, 113 genera, and 146 species were identified in the southern Altai Mountains. A total of six phytoplankton families, 66 genera, and 131 phytoplankton species were identified in the artificial water in the northern part of the Tianshan Mountains. A total of seven phytoplankton families, 58 genera, and 121 phytoplankton species were identified in artificial water in the southern part of the Tianshan Mountains (Table S1). Among them, the NT and ST had the highest number of overlapping phytoplankton species with 36 species, followed by the SA and NT with 17 species (Figure 3).

**Figure 4:**
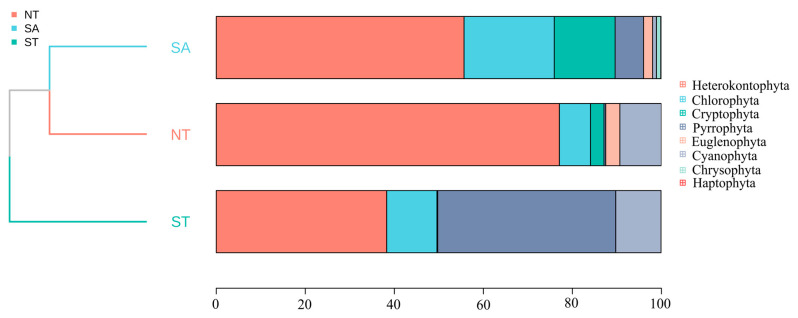
Percentage of phytoplankton accumulation and clustering analysis in different geographic settings.

The average biomass of the SA phytoplankton was 2.34 ± 0.60 mg/L and the average density was 5,364,948.42 ± 1,763,474.55 cells/L; the average biomass of the ST phytoplankton was 0.07 ± 0.01 mg/L and the average density was 140,876.01 ± 17,322.17 cells/L; and the average biomass of the NT phytoplankton was 0.13 ± 0.04 mg/L and the average density was 461,002.07 ± 131,938.61 cells/L. Across all three areas, diatom species contributed the most to the phytoplankton community biomass. The biomass percentage of Chlorophyta was highest in the SA compared to the other two areas, whereas in the ST, Chlorophyta had a higher overall percentage relative to the other areas. A cluster analysis indicated that the SA was most closely related to the NT (Figure 4).

**Figure 5:**
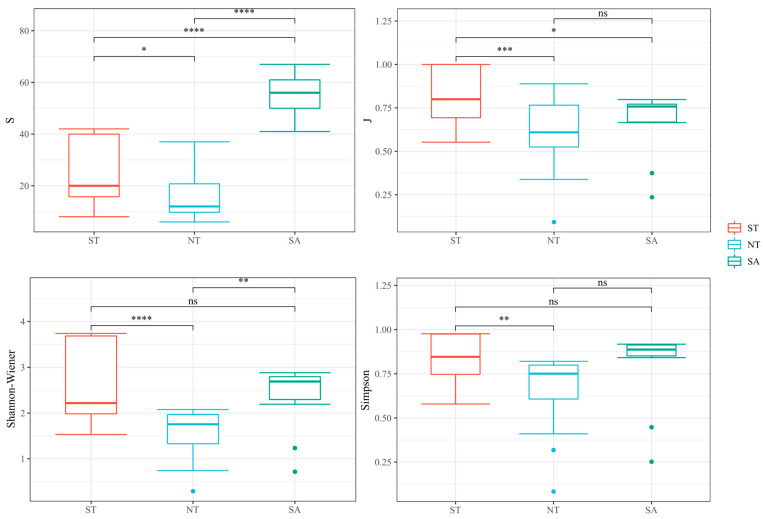
Analysis of phytoplankton diversity in different geographical environments, ns as no significance, * Benjamini-Hochberg adjusted 0.01 ≤ *p* < 0.05; ** Benjamini-Hochberg adjusted 0.001 ≤ *p* < 0.01; *** Benjamini-Hochberg adjusted *p* < 0.001; **** Benjamini-Hochberg adjusted *p* < 0.0001.

In several species, the SA significantly differed from both the ST and NT and substantially differed from the ST and NT. In terms of the Pielou index, the ST treatment significantly differed from the NT and SA treatments. In terms of the Shannon–Wiener index, the ST differed substantially from the NT, and the SA significantly differed from the NT. There was only a significant difference in the Simpson’s diversity index between the ST and NT treatments (Figure 5).

### 3.3. Interactions between Climate and Environmental Factors

The ANOSIM results showed that the R value of 0.96 for the NT and SA was large, with a *p* value of 0.001, indicating that the structural composition of the phytoplankton communities differed significantly between the NT and SA. The R value of 0.11 for the NT and ST was smaller, with a *p* value of 0.01, indicating that the structural composition of the phytoplankton communities differed between the NT and ST. The R value of 0.67 for the ST and SA was smaller, with a *p* value of 0.001, indicating that the structural composition of the phytoplankton communities differed between the NT and SA. The R value of 0.67 for the NT and ST, with a *p* value of 0.001, showed that the structural composition of the phytoplankton community differed between the ST and SA (Table 2).

**Figure 6:**
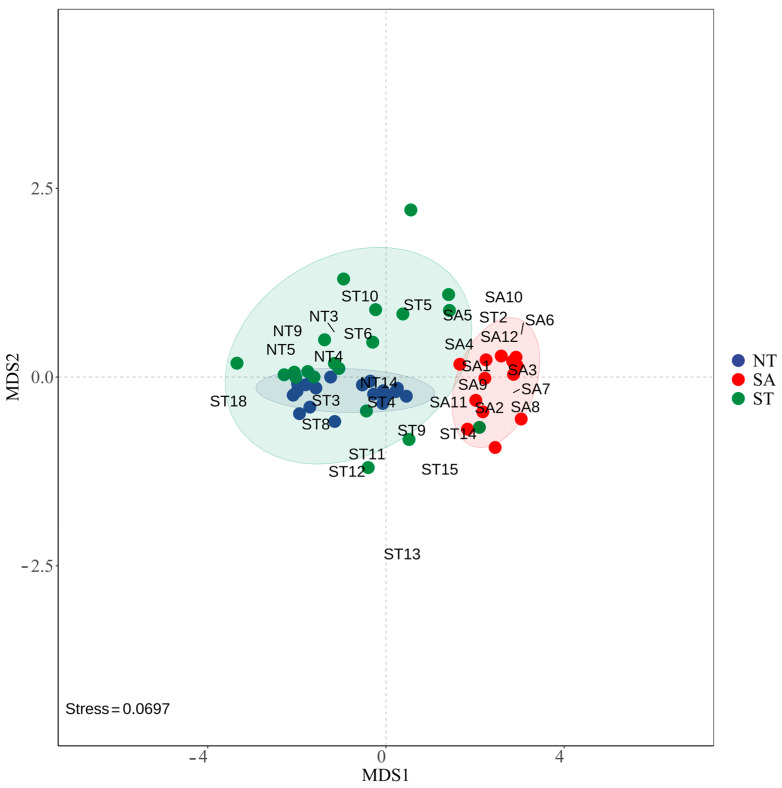
NMDS modeling of phytoplankton in different geographic environments.

The NMDS results showed that the phytoplankton communities were more similar between the ST and NT, while the SA community was farther from the other two communities, indicating that they were more different. The stress value of 0.0697 indicated that the NMDS model fit the raw data well and could accurately reflect the similarities or differences between samples (Figure 6).

**Figure 7:**
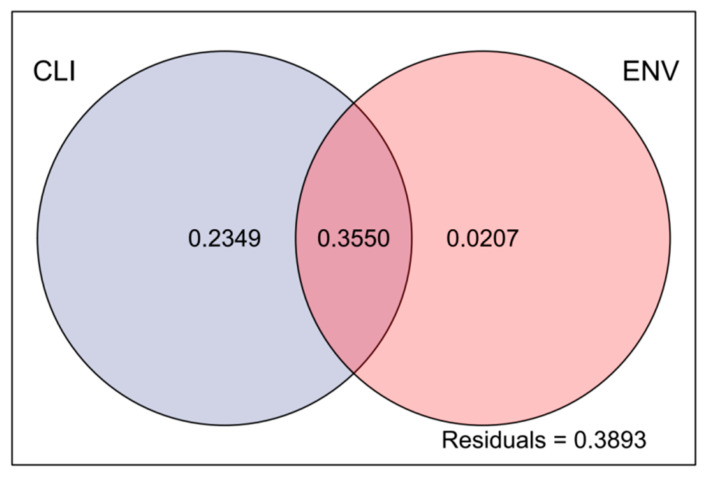
Analysis of the effects of climatic and environmental factors on phytoplankton communities. Abbreviations: CLI, climatic factors; ENV, environmental factors.

The VPA results showed that the total explained variance was 61.06% considering all the impact factors, indicating that the impact factors partially clarified the variation in the observed data, with climate factors explaining 23.49% and environmental factors explaining only 2.07% of the total variation. There was some overlap between the ecological and climate factors, which together explained 35.50% of the total variation, indicating an interaction between them. The residual value of 38.93% may have been influenced by other unmeasured factors or stochasticity (Figure 7).

## 4. Discussion

### 4.1. Correlation Analyses between the State of Watershed Ecosystems and Water Environment Parameters

In this study, we examined the artificial water environments in Central Asia to understand the spatial variability of water environment indicators across different regions and analyzed the resultant effects on phytoplankton growth, taking into account climatic data and geographic barriers. In the southern Altai Mountains, the range of water temperature variations was greater, and the variations in dissolved oxygen, total nitrogen, and total phosphorus were also greater; these physical and nutrient indicators have important effects on phytoplankton growth [39]. High variability in dissolved oxygen and nutrients (total nitrogen and total phosphorus) may lead to crucial changes in phytoplankton growth rates and community structure [40].

In contrast, artificial water bodies in the northern and southern Tianshan Mountains showed different characteristics. In the northern Tianshan Mountains, the water temperature was high, the range of variation in dissolved oxygen and nutrient salts was relatively small (Table 3), and the nutrient indicators were not significant, which may imply that the phytoplankton growth in this region is relatively stable and not easily affected by extreme environmental changes [41]. This indicates that phytoplankton growth in this region is more critically affected by water temperature and nutrient salts.

Combined with the analyses of climatic data, environmental factors such as the mean annual air temperature, temperature in the hottest and coldest months, annual precipitation, and seasonal variations crucially affect phytoplankton growth and reproduction. The SA have the lowest mean annual air temperature, the lowest temperature in the coldest month, and the highest annual precipitation, which are the conditions that limit the rapid growth of phytoplankton, but the nutrients generated by the abundant precipitation provide conditions for its growth [42,43,44]. The relatively warm mean annual temperatures and the high hottest month temperatures in the ST and NT regions promote phytoplankton colonization in summer, but the low precipitation and seasonal variations in the ST region may lead to unstable phytoplankton growth environments [45].

The accelerated melting of snow and ice in the ST due to global warming significantly impacts phytoplankton by altering environmental conditions and nutrient availability, affecting their growth and community dynamics [46]. Melting snow and ice alter environmental conditions and nutrient availability, which in turn affect phytoplankton growth [47]. Light-absorbing impurities in snow accelerate the melting process, affecting the timing and rate of snow and ice melt in the region [48]. This leads to changes in nutrient and light availability for phytoplankton, which may affect their growth and distribution [49].

Overall, variations in physical and nutrient indicators across regions significantly influence the ecological distribution and population dynamics of phytoplankton, with geographic barriers such as the Altai and Tianshan mountains playing a crucial role in these processes. High temperatures and sufficient nutrients promote phytoplankton growth, while temperature extremes and erratic precipitation may limit growth or lead to growth fluctuations [50]. These findings are important for understanding the dynamics of artificial water ecosystems and phytoplankton adaptation mechanisms in Central Asia.

### 4.2. Effects of Topographic Barriers on Phytoplankton Communities

The results revealed significant differences in the species composition, biomass, and density of phytoplankton communities among the SA, NT, and ST artificial waters. These differences are attributed to geographic location, climatic conditions, and nutrient status, which alter water dynamics, light availability, and nutrient transport. Geographical location and climatic conditions significantly influence phytoplankton communities [51]. Beyond a mild climate, factors like precipitation and seasonal variations impact nutrient inputs and water levels, subsequently affecting phytoplankton growth and distribution [52]. Our data showed that regions with higher precipitation and seasonal variation, such as the southern Altai Mountains, supported more robust phytoplankton blooms. The southern Altai Mountains receive more precipitation and have pronounced seasonal variations, providing ample water and nutrients that promote phytoplankton blooms [53]. Conversely, the northern and southern Tianshan Mountains experience less precipitation and seasonal variation, resulting in nutrient-poor waters that limit phytoplankton growth [54]. Our study confirmed these patterns, with higher phytoplankton diversity and density observed in the southern Altai region.

Climatic conditions affect phytoplankton growth and distribution by influencing water column circulation and mixing. Mild climates promote active water circulation, ensuring even nutrient distribution and supporting phytoplankton growth [50]. Our results showed that areas with more stable climatic conditions had more uniform phytoplankton distributions. Harsh climatic conditions can restrict water circulation, causing uneven nutrient distribution and limiting phytoplankton growth. Additionally, climatic factors affect water quality, including clarity and dissolved oxygen levels, which are crucial for phytoplankton adaptation and development. For instance, our study observed lower phytoplankton diversity in regions with more extreme climatic conditions. Under mild climatic conditions, water clarity will increase, and the dissolved oxygen content may increase, favoring phytoplankton photosynthesis and growth [55]. Conversely, adverse conditions result in turbid water and low oxygen levels, restricting phytoplankton growth [56]. Climatic conditions may also affect the structure and activity of microbial communities in the water column, affecting the ecology of phytoplankton. Under mild climatic conditions, microorganisms in the water column may be relatively abundant and diverse, forming a complex ecological network with phytoplankton and promoting phytoplankton growth and reproduction [57]. Under harsh climatic conditions, microorganisms in the water column may be increasingly less active, limiting the ecological environment and growth conditions for phytoplankton [58].

Regarding climatic factors, several studies have shown that the impact of climate change on watershed ecosystems is critical. Researchers have noted that the impacts of climate change on watershed ecosystems are multifaceted, with factors such as precipitation, temperature, and light being critical [59].

As an essential climate component, precipitation directly affects water levels and quality. Adequate precipitation helps to replenish the water volume and maintain the appropriate water level. Additionally, it helps dilute pollutants in the water body, provides an adequate supply of nutrients, and promotes phytoplankton growth [60]. In contrast, a decrease in precipitation under drought conditions may lead to a reduction in water level, eutrophication of the water body, and ecosystem collapse, limiting the growth and reproduction of phytoplankton [61].

Moreover, the water temperature is low under arid and cold climatic conditions, which may limit phytoplankton growth rates and ecological adaptations [62]. Adequate light facilitates plant photosynthesis, providing energy and nutrients that promote phytoplankton growth. In warm and humid climates, sufficient light favors photosynthesis and the development of phytoplankton. In contrast, light may be more limited at dry and cold temperatures, limiting phytoplankton growth [63].

The topographic barriers of the Altai and Tianshan mountain ranges also impact phytoplankton communities in the waters. These mountain ranges form a geographic barrier that affects the transport and distribution of nutrients in the water column. As two critical geographic barriers in Xinjiang, the Altai and Tianshan mountain ranges not only block climate exchange and hydrological connectivity between the two regions geographically but also influence atmospheric currents greatly, further shaping the distinct climatic and ecosystem patterns on both sides of the ranges [64]. The Altai Mountains are in the northern part of the Xinjiang region, and as part of Central Asia, they are bordered by Mongolia and Russia to the north. This location causes the Altai Mountains to be a crucial geographic barrier to the northern Asian continent, blocking cold air currents and weather systems from the north and keeping the climate on the southern flanks relatively warm and humid [65].

In contrast, the Tianshan Mountains are in the center of the Xinjiang region and run east–west across the region, dividing it into the northern and southern regions. The presence of the Tianshan Mountains also blocks atmospheric currents, limiting airflow from the western and eastern regions to the north and south of the mountains, resulting in a large difference in climate between the northern and southern regions [66]. This climatic difference directly affects the formation and distribution of phytoplankton communities. The different climatic conditions in the southern Altai Mountains and the northern and southern Tianshan Mountains cause the water environment to differ in temperature, humidity, and light, affecting phytoplankton growth, reproduction, and distribution [67]. For example, the relatively warmer and wetter southern side may be more suitable for developing certain phytoplankton (such as *Nitzschia Hassall*). In comparison, the relatively colder and drier northern side may limit the reproduction and distribution of certain phytoplankton (such as Bacillariophyta) [68]. Thus, the geographical position of the Altai and Tianshan mountain ranges not only produces differences in climate and ecosystems in terms of topography but also influences the movement and distribution of atmospheric currents, which further exacerbates the climatic differences between the areas on either side of the ranges, thus crucially impacting the formation and structure of phytoplankton communities [69].

Researchers have found large differences in artificial waters in different geographical locations and water body types, which directly affect the composition and abundance of phytoplankton communities. For example, a comparative study of mountainous and plain areas revealed that phytoplankton species richness was greater in mountainous water bodies, which may have been influenced by topographic and climatic conditions that elevate their species diversity [41]. Battegazzore conducted a study on alpine springs in NW Italy, indicating distinct environmental features based on prevalent diatom species in mountainous and lowland plain areas [70]. In contrast, water bodies in the plains were dominated by cyanobacteria and green algal species, which may have been influenced by human activities and environmental stresses, leading to an increase in eutrophication of the water bodies, which altered the structure and abundance of the phytoplankton communities [40].

The Altai Mountains, located in the easternmost Gobi Altai region, have experienced late Cenozoic transpressional deformation due to intracontinental mountain building processes [71]. The Tianshan Mountains, Tarim Basin, and Kunlun Mountains in Xinjiang have been affected by variability in supply and demand, with extensive deserts covering the region [72]. The foothills of the Altai and Tianshan mountains in Central Asia have also been impacted by arid zones, affecting the phytoplankton community in the region. The Tianshan Mountains of Northern China and Kyrgyzstan, which are critical sources of water, have experienced a decline in glaciers over the past 50 years, further emphasizing the need to address the impacts of human activities on the environment [73]. The Altai and inner Tianshan glaciers have been particularly affected by climatic warming and increasing anthropogenic activities, leading to changes in the phytoplankton community in the region [74].

### 4.3. Impacts of Climate Change on Alpine Aquatic Ecosystems

The ANOSIM results revealed significant differences in the phytoplankton community structures across artificial water bodies in different geographic locations, consistent with previous findings. For example, it was reported that differences in geographic location crucially affect the composition and abundance of phytoplankton communities in lakes with different geographic locations [75]. In addition, topography and geographic barriers also greatly impact the formation of biological communities in waters. The Altai Mountains and Tianshan Mountains, as geographic barriers, may have blocked the exchange of phytoplankton in the water bodies of different regions to some extent, which has led to the differentiation of phytoplankton communities in the NT and SA water bodies.

The NMDS results showed that the phytoplankton communities are more similar between the ST and NT treatments. In contrast, the SA group is more distant from the other two groups, implying that they are more differentiated. For example, there are large differences in phytoplankton community structure between waters, especially in areas more affected by human activities [76]. This study further supports our interpretation of the NMDS results. Various factors, including geographic location, hydrological conditions, nutrient status of the water body, and human activities, may influence this variability. In the southern region, human activities may have led to increased eutrophication of water bodies, which affects the structure and composition of phytoplankton communities, causing a difference from other regions. In addition, topographic barriers such as those in the Altai Mountains and the Tianshan Mountains may have blocked phytoplankton migration and exchange between areas, further exacerbating regional community differences.

Climatic factors such as temperature, precipitation, and light have multiple effects on watershed ecosystems, and they directly or indirectly influence the change in dynamics of phytoplankton communities. However, environmental factors such as the degree of eutrophication of water bodies and the level of pollutants may also affect the growth and distribution of phytoplankton [77]. In the southern region, human activities such as urban discharge and agriculture might have led to increased eutrophication, affecting phytoplankton community structures. Additionally, topographic barriers may hinder phytoplankton migration between regions, further accentuating community differences. Looking ahead, global warming could accelerate snow and ice melting in alpine regions, intensifying its impact on watershed ecosystems. Therefore, it is crucial to conduct in-depth studies on the effects of global warming on alpine aquatic ecosystems and implement appropriate measures to address this challenge.

## 5. Conclusions

Studies have shown that there are large differences in the physical indicators and water quality characteristics between the artificial water bodies in the southern Altai Mountains and those in the northern and southern Tianshan Mountains. These differences are mostly influenced by geographic location, climatic conditions, degree of eutrophication of water bodies, and topographic barriers. Geographic location and climatic conditions affect the formation and development of watershed ecosystems and physical indicators such as water body temperature and dissolved oxygen content. Topographic barriers such as the Altai and Tianshan mountain ranges may impede phytoplankton migration and exchange in water bodies in different regions, exacerbating differences in communities between regions. The results of multivariate analyses further confirmed the large differences in phytoplankton community structure in artificial water bodies in different geographical locations, which is consistent with the results of previous studies. In summary, this study provides insights into the effects of geographic location, climatic conditions, water body nutrient status, and topographic barriers on water body ecosystems and phytoplankton communities, which provides an important reference for better understanding the dynamics of aquatic ecosystems and provides a scientific basis for related environmental protection and ecological restoration. Future studies could further explore the interaction mechanisms between factors and their impacts on the stability and functioning of aquatic ecosystems to provide more effective conservation and management measures.

## Figures and Tables

**Table 1:** Average climate factors for the three regions.

Variable	SA	ST	NT
BIO1 ^1^	4.08	11.34	8.50
BIO2 ^2^	12.42	15.53	10.83
BIO3 ^3^	24.13	32.26	21.00
BIO4 ^4^	1458.27	1212.16	1519.32
BIO5 ^5^	28.98	33.13	32.16
BIO6 ^6^	−22.46	−14.93	−19.41
BIO7 ^7^	51.44	48.05	51.57
BIO8 ^8^	19.96	24.60	23.61
BIO9 ^9^	−12.22	-3.65	−10.80
BIO10 ^10^	20.83	24.60	25.11
BIO11 ^11^	−14.88	−4.81	−12.15
BIO12 ^12^	175.15	51.75	144.25
BIO13 ^13^	27.31	11.50	20.75
BIO14 ^14^	6.31	0.50	5.00
BIO15 ^15^	38.08	75.01	45.38
BIO16 ^16^	63.15	29.50	59.85
BIO17 ^17^	21.85	2.25	16.60
BIO18 ^18^	62.85	29.50	55.00
BIO19 ^19^	26.69	2.75	16.60

^1^ Annual mean temperature; ^2^ Mean diurnal range (monthly mean (max temp–min temp)); ^3^ Isothermality (BIO2/BIO7) (×100); ^4^ Temperature seasonality (standard deviation ×100); ^5^ Max temperature of warmest month; ^6^ Min temperature of coldest month; ^7^ Annual temperature range (BIO5-BIO6); ^8^ Mean temperature of wettest quarter; ^9^ Mean temperature of driest quarter; ^10^ Mean temperature of warmest quarter; ^11^ Mean temperature of coldest quarter; ^12^ Annual precipitation; ^13^ Precipitation of wettest month; ^14^ Precipitation of driest month; ^15^ Precipitation seasonality (Coefficient of Variation); ^16^ Precipitation of wettest quarter; ^17^ Precipitation of driest quarter; ^18^ Precipitation of warmest quarter; ^19^ Precipitation of coldest quarter.

**Table 2:** Analysis of differences in phytoplankton structure in different geographic environments.

Group	R ^1^	*p* ^2^
NT/SA	0.964	0.001 **
NT/ST	0.110	0.010 *
SA/ST	0.673	0.001 **

^1^ R ranges from −1 to 1, with 0 indicating no similarity among groups and 1 indicating maximum similarity. ^2^ ** = *p* < 0.01; * = *p* < 0.05.

**Table 3:** Physical and nutritional parameters in three areas.

		Physical							Nutritional	
		WT (°C)	ORP (mv)	pH	DO (mg/L)	C (μS/cm)	SAL (ppt)	TDS (mg/L)	TN (mg/L)	TP (mg/L)
SA	average	21.98	91.77	8.72	5.22	416.66	0.21	0.29	0.87	0.64
	standard	4.11	53.46	0.22	1.99	296.36	0.15	0.19	0.32	0.24
ST	average	12.77	96.55	7.60	5.85	328.35	0.08	222.25	0.04	0.15
	standard	7.17	25.95	0.25	2.30	77.84	0.07	164.19	0.02	0.02
NT	average	23.40	91.87	7.80	7.28	228.65	0.06	131.50	0.58	0.83
	standard	3.68	26.61	0.34	1.81	57.01	0.03	53.76	0.13	0.14

## Data Availability

The data supporting this study’s findings are available from the corresponding authors upon reasonable request.

## Supplementary Materials

The supporting information can be downloaded at https://www.mdpi.com/article/10.3390/biology13090717/s1, Table S1: Phytoplankton species list.
