# Supplementary material for: Environmental and Climatic Drivers of Phytoplankton Communities in Central Asia
Source: Biology (Basel). 2024 Sep 12;13(9):717. doi: 10.3390/biology13090717 (PMC11428709; doi:10.3390/biology13090717)
Supplement: Supplementary file 1 [file biology-13-00717-s001.zip › biology-3175221-supplementary.pdf]

Table S1. Phytoplankton species list.

| SA | NT | ST |
|---|---|---|
| *Microcystis* sp. | *Cryptomonas ovata* | *Denticula kuetzingii* |
| *Chroococcus* sp. | *Cyclotella meneghiniana* | *Cocconeis placentula* var. *lineata* |
| *Aphanocapsa* sp. | *Strombomonas acus* | *Achnanthes exigua* |
| *Merismopedia* sp. | *Strombomonas rotunda* | *Cymbella* sp. |
| *Rhabdogloea* sp. | *Nitzschia palea* | *Amphora ovalis* |
| *Dactylococcopsis* sp. | *Euglena viridis* | *Navicula exigua* |
| *Gomphosphaeria* sp. | *Melosira granulata* | *Fragiaria construens* |
| *Woronichinia* sp. | *Nitzschia acuta* | *Cyclotella meneghiniana* |
| *Lyngbya* sp. | *Cryptomonas erosa* | *Melosira ambigua* |
| *Hydrocoleum* sp. | Melosira granulata var. angustissima | *Pteromonas angulosa* |
| *Planktothrix* sp. | *Pediastrum duplex* var. *reticulatum* | *Cryptomonas erosa* |
| *Oscillatoria* sp. | *Ankistrodesmus acicularis* | *Fragilaria brevistriate* |
| *Spirulina* sp. | *Synedra acus* | *Limnothrix planctonica* |
| *Gloeotrichia* sp. | *Actinastrum fluviatile* | *Aphanothece clathrata* |
| *Limnothrix* sp. | *Chlamydomonas globosa* | *Chroococcus minutus* |
| *Leptolyngbya* sp. | *Scenedesmus bicaudatus* | *Microcystis flos-aquae* |
| *Pseudoanabaena* sp. | *Chlorella vulgaris* | *Scenedesmus bijuga* |
| *Dolichospermum* sp. | *Navicula cincta* | *Chlamydomonas globosa* |
| *Melosira* sp. | *Synedra ulna* | *Monoraphidium contortum* |
| *Melosira granulata* | *Closterium gracile* | *Scenedesmus quadricauda* |
| *Melosira varians* | *Scenedesmus quadricauda* | *Nitzschia palea* |
| *Chaetoceros* sp. | *Oscillatoria fraca* | *Synedra acus* |
| *Coscinodiscus* sp. | *Lepocinclis ovum* | *Melosira granulata* |
| *Cyclotella* sp. | *Oocystis borgei* | *Pinnularia molaris* |
| *Attheya* sp. | *Nitzschia sinuata* | *Anabaena flos-aquae* |
| *Tabellaria* sp. | *Pseudanabaena limnetica* | *Nitzschia angustata* |
| *Diatoma* sp. | *Navicula rhynchocephala* | *Chrysococcus rufescens* |
| *Meridion* sp. | *Nitzschia acicularis* | *Monoraphidium irregulare* |
| *Ceratoneis* sp. | *Pandorina morum* | *Kephyrion planctonicum* |
| *Fragilaria* sp. | *Thorakomonas sabulosa* | *Dictyosphaerium reniforme* |
| *Synedra* sp. | *Scenedesmus bijuga* | *Cymatopleura solea* |
| *Synedra ulna* | *Chroomonas acuta* | *Navicula cryptocephala* |
| *Asterionella* sp. | *Raphidiopsis curvata* | *Nitzschia denticula* |
| *Asterionella glacialis* | *Quadricoccus alternans* | *Cymbella cistula* |
| *Mastogloia* sp. | *Scenedesmus spinosus* | *Navicula capitata* |
| *Frustulia* sp. | *Tetrastrum multiasetum* | *Nitzschia amphibia* |
| *Gyrosigma* sp. | *Nitzschia closterium* | *Mougeotia quadrangulata* |
| *Caloneis* sp. | Chlamydomonas sp. | *Cymbella ventricosa* |
| *Diploneis* sp. | *Anabaena flos-aquae* | *Achnanthes minutissima* |
| *Stauroneis* sp. | *Chroococcus minutus* | *Navicula radiosa* |
| *Navicula* sp. | *Microcystis flos-aquae* | *Navicula minuscula* |
| *Pinnularia* sp. | *Kirchneriella lunaris* | *Surirella ovalis* |
| *Amphiprora* sp. | *Kirchneriella obesa* | *Navicula minima* |
| *Amphora* sp. | *Tetrastrum staurogeniaeforme* | *Pseudanabaena limnetica* |
| *Cymbella* sp. | *Ankistrodesmus falcatus* | *Pinnularia undulata* |

| | | |
|---|---|---|
| *Didymosphenia geminata* | *Monoraphidium contortum* | *Stauroneis anceps* |
| *Gomphonema* sp. | *Kirchneriella contorta* | *Dactylococcopsis rhaphiioides* |
| *Achnanthes* sp. | *Dactylococcopsis rhaphiioides* | *Eunotia arcus* |
| *Rhoicosphenia curvata* | *Lagerheimiella subsalsa* | *Cymbella turgida* |
| *Cocconeis* sp. | *Tetraedron pusillum* | *Nitzschia sigma* |
| *Epithemia* sp. | *Tetraedron trigonum* | *Cymbella ehrenbergii* |
| *Rhopalodia* sp. | *Merismopedia tenuissima* | *Nitzschia acicularis* |
| *Rhopalodia gibba* | *Nitzschia paradoxa* | *Peridiniopsis niei* |
| *Hantzschia* sp. | *Surirella ovata* | *Dinobryon cylindricum* |
| *Nitzschia* sp. | *Achnanthes exigua* | *Ankistrodesmus acicularis* |
| *Nitzschia paradoxa* | *Gyrosigma attenuatum* | *Nitzschia obtusa* |
| *Surirella* sp. | *Amphiprora alata* | *Ankistrodesmus angustus* |
| *Surirella capronii* | *Diploneis oblongella* | *Pseudanabaena minima* |
| *Cymatopleura solea* | *Navicula exigua* | *Gyrosigma kuetzingii* |
| *Cymatopleura elliptica* | *Navicula minima* | *Cymatopleura elliptica* |
| *Chlamydomonas* sp. | *Oscillatoria subbrevis* | *Chroomonas caudata* |
| *Phacotus* sp. | *Navicula capitata* | Pediastrum simplex var. sturmii |
| *Pyramimonas* sp. | *Oscillatoria chlorina* | *Merismopedia sinica* |
| *Pteromonas* sp. | *Phacus platyaulax* | *Crucigenia tetrapedia* |
| *Gonium* sp. | *Golenkinia radiata* | *Ankistrodesmus falcatus* |
| *Eudorina elegans* | *Dictyosphaerium pulchellum* | *Stauroneis palustris* |
| *Pandorina morum* | *Tetraedron caudatum* | *Merismopedia tenuissima* |
| *Asterococcus* sp. | *Diatoma hiemale* | *Oscillatoria fraca* |
| *Tetraspora* sp. | *Nitzschia panduriformis* | *Gymnodinium aeruginosum* |
| *Elakatothrix gelatinosa* | *Surirella ovata* var. *salina* | *Tetrastrum punctatum* |
| *Micractinium* sp. | *Amphora ovalis* | *Dinobryon divergens* |
| *Golenkinia* sp. | *Diploneis finica* | *Tetraedron pusillum* |
| *Characium* sp. | *Navicula ammophila* var. *minuta* | *Oocystis lacustris* |
| *Schroederia* sp. | *Pseudanabaena catenata* | *Gymnodinium mitratum* |
| *Chodatella* sp. | *Monoraphidium griffithii* | *Scenedesmus acuminatus* |
| *Chodatella quadriseta* | *Cymbella hybrida* | *Ceratium hirundinella* |
| *Franceia ovalis* | *Gyrosigma scalproides* | *Aphanocapsa delicatissima* |
| *Tetraëdron caudatum* | Navicula protracta var. elliptica | *Achnanthes linearis* |
| *Tetraëdron trilobulatum* | *Diatoma moniliforme* | *Mastogloia smithii* |
| *Tetraëdron minimum* | *Stephanodicus minutulus* | *Scenedesmus bicaudatus* |
| *Tetraëdron trigonum* | *Anabaenopsis arnoldii* | *Merismopedia punctata* |
| *Ankistrodesmus* sp. | *Aphanocapsa delicatissima* | *Monoraphidium mirabile* |
| *Selenastrum* sp. | *Treubaria crassispina* | *Diploneis ovalis* |
| *Kirchneriella* sp. | *Monoraphidium circinale* | *Navicula simplex* |
| *Treubaria crassispina* | *Schroedria setigera* | *Scenedesmus ankistrodesmoides* |
| *Sphaercystis* sp. | *Schroedria spiralis* | Chlorella vulgris |
| *Oocystis* sp. | *Coelastrum microporum* | *Chroomonas acuta* |
| *Nephrocytium* sp. | *Crucigenia quadrata* | *Spirogyra catanaeformis* |
| *Dictyosphaerium* sp. | *Scenedesmus acuminatus* | *Oocystis parva* |
| *Pediastrum* sp. | *Peridiniopsis elpatiewskyi* | *Peridiniopsis cunningtonii* |

| | | |
|---|---|---|
| *Pediastrum simplex* var. *duodenarium* | *Merismopedia minima* | *Cyclotella stelligera* |
| *Pediastrum duplex* | *Chroococcus minor* | *Limnothrix redekei* |
| *Pediastrum boryanum* | *Oocystis lacustris* | *Closterium kuetzingii* |
| *Pediastrum tetras* | *Oocystis parva* | *Oocystis borgei* |
| *Scenedesmus* sp. | *Rhoicosphenia curvata* | *Rhopalodia gibba* |
| *Scenedesmus bijugus* | *Fragilaria brevistriate* | *Stauroneis phoenicenteron* |
| *Scenedesmus bicaudatus* | *Pseudanabaena mucicola* | *Chroococcus turgidus* |
| *Scenedesmus quadricauda* | *Anabaena eucompaeta* | *Trachelomonas volvocina* |
| *Scenedesmus dimorphus* | *Phacus agilis* | *Cocconeis placentula* |
| *Scenedesmus arcuatus* | *Trachelomonas volvocina* | *Chlamydomonas microsphaerella* |
| *Scenedesmus acuminatus* | *Gloeoactiniuum limneticum* | *Leptolyngbya valderiana* |
| *Scenedesmus obliquus* | *Monoraphidium caribeum* | *Epithemia sorex* |
| *Tetrastrum* sp. | *Crucigenia apiculata* | *Leptolyngbya valderiana* |
| *Tetrastrum staurogeniaeforme* | *Tetrastrum glabrum* | *Ceratium furcoides* |
| *Crucigenia* sp. | *Scenedesmus denticulatus* | *Scendesmus ecornis* |
| *Crucigenia apiculata* | Cocconeis placentula var. lineata | *Merismopedia minima* |
| *Crucigenia tetrapedia* | *Chroococcus limneticus* | *Fragilaria pinnata* |
| *Crucigenia quadrata* | *Phacus bacilliformis* | *Cymbella parva* |
| *Actinastrum* sp. | *Trachelomonas spinulosa* | *Crucigenia rectangularis* |
| *Coelastrum* sp. | *Treubaria triappendiculata* | *Oedogonium pusillum* |
| *Coelastrum reticulatum* | Tetraedron caudatum var. longispinum | *Asterionella formosa* |
| *Closterium* sp. | *Merismopedia sinica* | *Oscillatoria animalis* |
| *Closterium gracile* | *Tetraedron regulare* var. *incus* | *Gomphonema intricatum* |
| *Cosmarium* sp. | *Coelastrum sphaericum* | *Pseudanabaena catenata* |
| *Staurastrum* sp. | *Crucigenia divergens* | *Synedra ulna* |
| *Euastrum* sp. | *Scenedesmus cavinatus* | *Scendesmus wuhanensis* |
| *Spondylosium* sp. | *Dysmorphococcus variabilis* | *Cymbella cuspidata* |
| *Gonatozygon* sp. | *Phacus pyrum* | *Peridinium gatunense* |
| *Ulothrix* sp. | *Phacus stokesii* | *Snowella lacustris* |
| *Klebsormidium* sp. | *Euglena pisciformis* | *Pediastrum duplex* |
| *Geminella* sp. | *Nephrocytium agardhianum* | *Scenedesmus aldavei* |
| *Planctonema* sp. | *Chlorococcum humicola* | |
| *Zygnema* sp. | *Tetrastrum punctatum* | |
| *Spirogyra* sp. | *Gloeocystis ampla* | |
| *Mougeotia* sp. | *Oocystis solitaria* | |
| *Cladophora* sp. | *Pediastrum tetras* | |
| *Oedogonium* sp. | *Scenedesmus protuberans* | |
| *Botryococcus braunii* | *Pinnularis viridis* | |
| *Chroomonas* sp. | *Snowella lacustris* | |
| *Chroomonas acuta* | *Gloeocystis vesiculosa* | |
| *Cryptomonas* sp. | *Westella botryoides* | |
| *Cryptomonas ovata* | | |
| *Euglena* sp. | | |
| *Euglena acus* | | |
| *Phacus* sp. | | |
| *Phacus agilis* | | |

| |
|---|
| *Trachelomonas* sp. |
| *Peridinium* sp. |
| *Gymnodinium* sp. |
| *Ceratium* sp. |
| *Dinobryon* sp. |
| *Kephyrion* sp. |
| *Chrysococcus* sp. |
| *Synura* sp. |
| *Mallomonas* sp. |
| *Chrysochromulina parva* |
